# Supplementary material for: Anti-SARS-CoV-2 Agents in Artemisia Endophytic Fungi and Their Abundance in Artemisia vulgaris Tissue
Source: J Fungi (Basel). 2023 Sep 5;9(9):905. doi: 10.3390/jof9090905 (PMC10532694; doi:10.3390/jof9090905)
Supplement: Supplementary file 1 [file jof-09-00905-s001.zip › jof-2569432-supplementary.pdf]

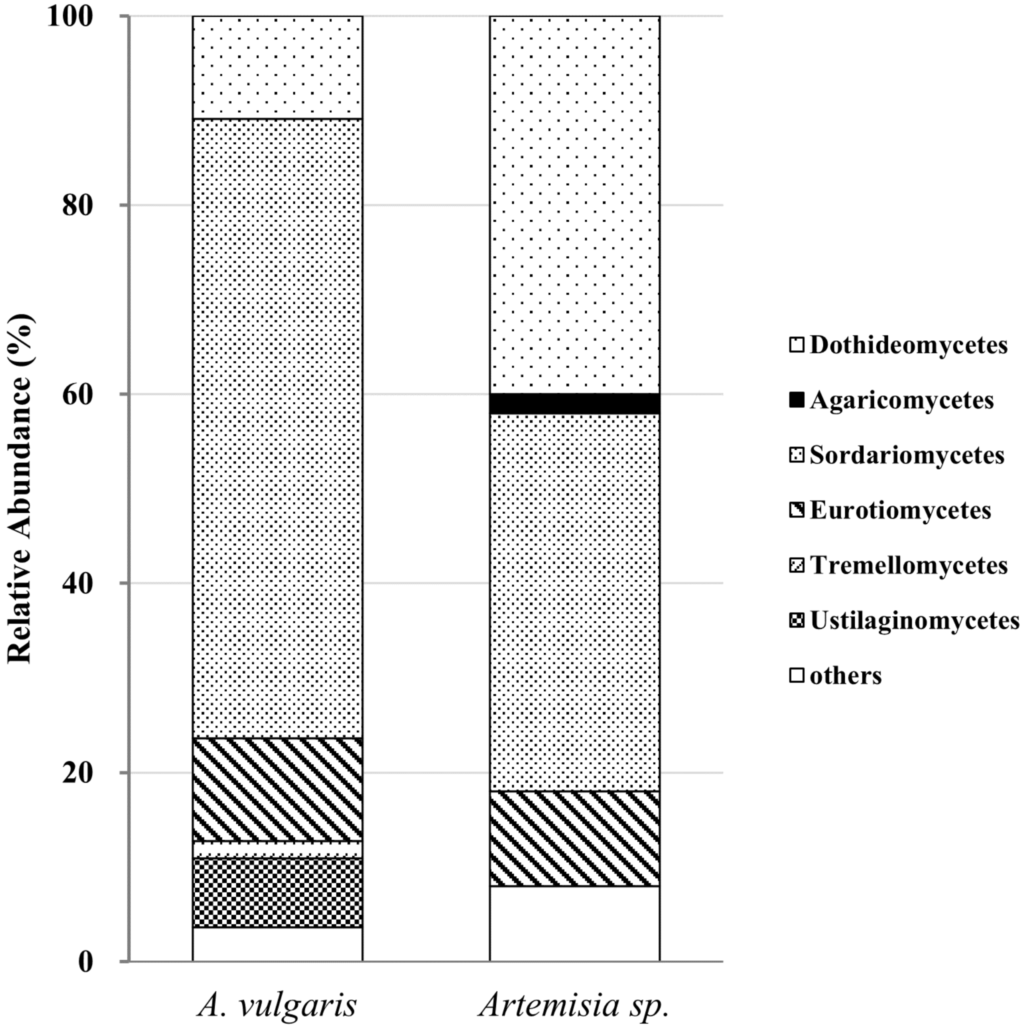

Figure S1. Class composition of endophytic fungi from isolated data of *A. vulgaris* [14] and *Artemisia* sp. [13]

[14] *J. Nat. Med.* **2023**, doi:10.1007/S11418-023-01709-7.

[13] *J. Fungi* **2018**, *4*, 53. https://doi.org/10.3390/jof4020053
